# Association between nurse turnover and missed nursing care in acute care hospitals in South Korea

**DOI:** 10.3389/fmed.2024.1448839

**Published:** 2025-01-07

**Authors:** Sung-Heui Bae

**Affiliations:** College of Nursing, Graduate Program in System Health Science and Engineering, Ewha Womans University, Seoul, Republic of Korea

**Keywords:** nurse turnover, missed nursing care, administration, acute hospitals, evaluation, quantitative study

## Abstract

**Objectives:**

High nurse turnover during nursing shortages can contribute to missed nursing care. This study investigated the prevalence of missed nursing care and how nurse turnover affects missed nursing care.

**Methods:**

A cross-sectional design was adopted to collect data from a convenience sample of nurses working in general hospitals in South Korea. Six-month turnover rates (0%, 1–14%, 15–22%, and 23–50%) and 24 missed nursing care activities were measured. A multivariate regression analysis was performed to examine the relationship between nurse turnover and missed nursing care, after controlling for nurse and work-related characteristics.

**Results:**

The final sample was 264 nurses. The mean six-month turnover rate was 15.49%. Seven activities (turning patient every 2 h, attending interdisciplinary care conference, ambulation, patient bathing/skin care, emotional support, mouth care, full documentation) had a missed care prevalence of 30% or higher. Nurses in units with moderate turnover rates (15 and 22%) reported more missed nursing care than those in units with zero turnover.

**Conclusion:**

Nurse turnover increases missed nursing care, highlighting the adverse effects of nurse turnover on care processes. Consequently, hospitals and governments should implement policy changes and strategies to prevent nurse turnover.

## Introduction

Missed nursing care—the omission of the required care by nurses (1)—can be partial, complete, or delayed. When time is limited, nurses may simultaneously provide multiple types of care while omitting others (2). A review reported that at least 75% of nurses missed care (3). Missed nursing care is more likely in work environments with fewer resources, such as those with poor staffing levels (4–6). When nurses care for several patients, they are more likely to miss the necessary care activities as compared to their counterparts. Moreover, patient characteristics such as turnover and acuity are associated with increased missed nursing care (5).

Missed nursing care affects nurses’ job outcomes and patient outcomes. Higher levels of missed nursing care are related to lower job satisfaction and a greater intent to leave (2). When nurses encounter situations in which some required care was omitted, they may experience inner conflicts with professional standards (7), which can lead to job dissatisfaction (2). Further, they may want to leave their positions. Missed nursing care affects patient mortality (8), safety (2, 9), and patients’ satisfaction/dissatisfaction (10), thereby undermining the quality of patient care.

Male and female nurses report different levels of missed nursing care (11). Further, nurses with more work experience perceive higher levels of missed care than those with less work experience (11). Nurses’ workload (12, 13), overtime work (14) and inadequate staffing perceived by nurses (2, 15) also contribute to missed nursing care. Perceived staffing adequacy by both patients and nurses is negatively associated with missed nursing care, specifically concerning communication (16). A systematic review revealed that lower nurse staffing levels are associated with missed nursing care (3, 17). Hospital size and unit type are also associated with missed nursing care (2), and staffing levels might negatively affect nurses’ health and lead to missed nursing care (18). A previous study identified a correlation between presenteeism and missed nursing care, highlighting its significant impact of on quality of care (19). A recent study found a negative relationship between work environment and missed nursing care (14, 20). Further, a favorable nurse practice environment was related to a lower frequency of missed nursing care (21). Team work is another critical factor contributing to missed nursing care (22).

In South Korea, the nurse turnover rate was 12.4%, while that of newly licensed registered nurses was 42.7% (23). A recent study in South Korea (2) reported that nurses, on average, missing 8.9 of 24 nursing activities with 10 activities exhibiting a prevalence of missed care of 50% of higher (3). Such high nurse turnover can lead to longer working hours and a higher nurse-patient ratio. Consequently, nurses may want to leave their positions, perpetuating a nursing shortage-turnover cycle. Nurses working in units with high turnover rates could experience frequent adjustments and spend more time supervising new staff members (24). They may also need to care for more patients (25), potentially leading to teamwork deterioration (22) and difficulty in complying with patient safety guideline (21), resulting in frequently missed nursing care. Despite the apparent relationship between high turnover and missed nursing care, the relationship between nurse turnover and missed nursing care has received scant research attention. It is crucial to examine the relationship between nurse turnover and missed nursing care to elucidate the underlying mechanism of the impact of nurse turnover on care process.

### Aims

This study investigated the prevalence of missed nursing care and how nurse turnover affects missed nursing care. The findings provide information about the impact of nurse turnover on the care processes, which informs policies and strategies to prevent nurse turnover and missed nursing care.

## Methods

### Study design, sample, and data collection

This cross-sectional study examined the prevalence of missed nursing care and how nurse turnover affects missed nursing care. Convenience sampling was used to collect data from nurses working in medical and surgical nursing units of acute care hospitals. Data were collected between July and September 2022. Only nurses who provided direct nursing care and worked in the current unit for at least 6 months were included.

From the registry of hospitals in Korea, 270 general hospitals with 201–1,000 beds in South Korea were invited to participate. Each hospital received information about the current study. Among 270 general hospitals, 35 agreed to participate. An online survey was administered to nurses and nurse managers in these hospitals. In total, 397 nurses and nurse managers in 35 hospitals responded to the online survey. Based on the inclusion criteria in the current study, participants were excluded if they had worked in the current unit for less than 6 months (*n* = 29), had not worked in medical or surgical units (*n* = 45), failed to answer more than 70% of the survey questionnaires (*n* = 26), or were nurse managers (*n* = 33). The survey focused on missed nursing care of staff nurses who provide direct nursing care, therefore, it excluded nurse managers who do not fit this criterion. Thus, data were analyzed from 264 staff nurses. According to G*Power 3.1.9.4 (26), a minimum sample of 150 participants was required for a multivariate regression analysis with 18 predictors, an effect size of 0.15, a significance level of 0.05, and a power of 0.80. Therefore, the sample size was sufficient.

### Measures

#### Nurse turnover

Nurse turnover was measured using the six-month turnover rates in a nursing unit. Nurse managers provided the number of nurses who left their units between January 1, 2022, and June 30, 2022 (approximately 6 months prior to data collection). The six-month turnover rate was calculated as follows as given below, which was used a previous study (24).


$$\begin{array}{l} \mathrm{Six-month turnover rate}=\\ \frac{\mathrm{Number of nurses}\;\mathrm{who}\;\mathrm{resigned between January}\;1\;\mathrm{and June}\;30,2022\;\mathrm{in}\;a\;\mathrm{unit}}{\mathrm{Average number of nurses working between January}\;1\;\mathrm{and June}\;30,2022\;\mathrm{in}\;a\;\mathrm{unit}} \end{array}$$

This turnover rate was calculated for nurses working with nurse managers in the same unit. To distinguish low and high levels of nurse turnover, the distribution and levels of turnover were assessed. As a result, the six-month turnover rate was categorized into four groups: zero (0%), low (1–14%), moderate (15–22%), and high (23–50%).

#### Missed nursing care

The Missed Nursing Care Survey (1) comprising 24 nursing activities measures missed nursing care. The Korean version developed by Cho et al. (2) was used in this study. Nurses reported the frequency of missed nursing care for each activity as rarely (1 point), occasionally (2 points), frequently (3 points), always (4 points), or not applicable. Each nurse responded according to the care activities they missed. The prevalence of missed nursing care was measured considering the number of nurses who responded “occasionally” to “always” (the numerator) and the total number of nurses who responded, excluding “not applicable” responses (the denominator). The mean missed nursing care score was calculated by the averaging nurses’ responses to all 24 activities, excluding those marked as “not applicable.” Kalisch and Williams (1) tested its validity and reliability and reported psychometric standards of this instrument. Cronbach’s alpha of the Korean version of the Missed Nursing Care Survey in Cho’s et al. (2) study was 0.88, while in the current study it was 0.91.

#### Nurse and work-related characteristics

To examine the impact of nurse turnover on missed nursing care, this study accounted for several confounding variables, including nurse and work-related characteristics. Nurses’ characteristics included sex, age, highest level of nursing education (associate’s degree, bachelor degree, master’s degree or PhD in nursing), and subjective health status (very good, good, fair, poor). Work-related characteristics included work type (three-shift rotation vs. others), work experience in the current hospital, workload, perceived staffing adequacy, type of nursing unit, and hospital size (number of beds).

Workload was measured as the level of performance required for a job (27, 28). It comprised four items that assessed the amount, intensity, and frequency of work on a six-point Likert scale (from “never” to “five or more days a week”). Total scores ranged from 4 to 24. Higher scores indicated higher workload. Cronbach’s alpha in this study was 0.82, indicating good internal consistency.

Perceived staffing adequacy was measured by one item, which is asking nurses’ perception regarding staffing adequacy. It used a four-point Likert scale (very insufficient, insufficient, sufficient, very sufficient).

### Data analysis

Data analysis was conducted using (SAS 9.4, Cary, NC, United States). The means and standard deviations of the six-month turnover rates were evaluated and grouped as 0%, 1–14%, 15–22%, and 23–50%. Mean scores for missed nursing care and the prevalence of missed nursing care were calculated. Descriptive statistics for the nurse and work-related characteristics were obtained. Missed nursing care based on participants’ characteristics was evaluated by conducting *t*-tests and an analysis of variance. A multivariate regression analysis was performed to examine the relationship between nurse turnover and missed nursing care, after controlling for nurse and work-related characteristics. In the multivariate regression analysis, hospital size was divided by 10 for better interpretation. Owing to missing data, the sample sizes per variable varied.

### Ethics statement

An appropriate institutional review board approved this study (Approval No. XXX-202205-0005-01). Informed consent was obtained online from all participants. Permissions to use the study instruments were obtained from their respective authors.

## Results

### Characteristics of study variables

In total, data from 264 staff nurses was used in this study. Table 1 presents participants’ demographic and work-related characteristics. Nearly all (97%) participants were women. The mean age of participant was 32.58 (SD = 7.97) years. Approximately half were aged 21–30 years. Approximately 82% of participants had at least a bachelor’s degree, and 154 (66.6%) perceived their subjective health status as fair or poor. More than 90% worked in three-shift rotations. Participants had an average of 7.62 years of work experience in their current hospitals. The mean workload score was 18.05 points (SD = 3.60), and 208 (82.5%) participants reported either very insufficient or insufficient staffing adequacy.

**Table 1:** General characteristics of the participants and study variables (*N* = 264).

Variables	*n* (%)	Mean (SD)
Nurse and work-related characteristics		
Sex	*N* = 234	
Male	5 (2.1)	
Female	229 (97.9)	
Age, years in 2022	*N* = 231	32.58 (7.97)
21–30	121 (52.4)	
31–40	67 (29.0)	
41–50	32 (13.8)	
≥51	11 (4.8)	
Highest nursing education	*N* = 230	
Associate’s degree	41 (17.8)	
Bachelor’s degree	178 (77.4)	
Master’s degree or PhD in nursing	11 (4.8)	
Subjective health status	*N* = 231	
Very good	20 (8.7)	
Good	57 (24.7)	
Fair	111 (48.0)	
Poor	43 (18.6)	
Work type	*N* = 264	
3 shifts rotation	239 (90.5)	
Other	25 (9.5)	
Work experience in current hospitals	*N* = 234	7.62 (7.18)
Under 1 year	16 (6.8)	
1 year–under 3 years	54 (23.1)	
3 years–under 5 years	37 (15.8)	
5 years–under 10 years	60 (25.7)	
10 years or over	67 (28.6)	
Workload	*N* = 263	18.05 (3.60)
Perceived staffing adequacy	*N* = 252	1.74 (0.81)
Very insufficient	117 (46.4)	
Insufficient	91 (36.1)	
Sufficient	37 (14.7)	
Very sufficient	7 (2.8)	
Type of unit	*N* = 264	
Medical	77 (29.2)	
Surgical	97 (36.7)	
Medical-surgical	90 (34.1)	
Hospital size (beds)	*N* = 264	372.65 (115.03)
201–300	106 (40.1)	
301–400	49 (18.6)	
401–500	83 (31.4)	
501–1,000	26 (9.9)	
Nurse turnover		
Turnover rate for 6 months	*N* = 217	15.49 (14.20)
0%	53 (24.4)	
1–14%	50 (23.0)	
15–22%	60 (27.7)	
23–50%	54 (24.9)	
Missed nursing care (mean score)	*N* = 195	1.33 (0.33)

SD, standard deviation; PhD, Doctor of Philosophy.

The mean six-month turnover rate was 15.49% [standard deviation (SD) = 14.2]. Approximately 24% of participants worked in nursing units with a zero turnover rate. Sixty (27.7%) nurses worked in units with a 15–22% turnover rate. The mean score of missed nurse care ranged from 1 to 3 (mean = 1.33, SD = 0.33).

### Prevalence and nature of missed nursing care

Table 2 presents the details of missed nursing care. Seven activities had a missed care prevalence of 30% or higher. The top five activities with the highest prevalence were turning the patient every 2 h, attending interdisciplinary care conferences, ambulation, patient bathing/skin care, and emotional support. Seven nursing care activities had a higher proportion of “not applicable”: turning patients every 2 h, attending interdisciplinary care conferences, ambulation, patient bathing/skin care, mouth care, feeding patients, and setting up meals. Ten nursing activities had a missed care prevalence of less than 20%.

**Table 2:** Prevalence of missed nursing care and priority rank.

		Missed care *n* (%)					Prevalence of missed care	
	*N*	Rarely (1)	Occasionally (2)	Frequently (3)	Always (4)	Not applicable	%^a^	Rank
Turning patient every 2 h	196	58 (29.6)	73 (37.2)	27 (13.8)	5 (2.6)	33 (16.8)	64.4	1
Attending interdisciplinary care conference	195	58 (29.7)	59 (30.3)	18 (9.2)	7 (3.6)	53 (27.2)	59.2	2
Ambulation	196	59 (30.1)	63 (32.1)	19 (9.7)	3 (1.5)	52 (26.6)	59.0	3
Patient bathing/skin care	195	63 (32.3)	51 (26.1)	11 (5.6)	4 (2.1)	66 (33.9)	51.2	4
Emotional support	195	92 (47.2)	71 (36.4)	19 (9.7)	4 (2.1)	9 (4.6)	50.5	5
Mouth care	195	69 (35.4)	49 (25.1)	15 (7.7)	2 (1.0)	60 (30.8)	48.9	6
Full documentation	196	123 (62.8)	62 (31.6)	7 (3.6)	0 (0.0)	4 (2.0)	35.9	7
Assisting with toileting within 5 min of request	195	121 (62.1)	43 (22.1)	2 (1.0)	2 (1.0)	27 (13.8)	28.0	8
Feeding patient	195	101 (51.8)	24 (12.3)	10 (5.1)	2 (1.0)	58 (29.8)	26.3	9
Assessing effectiveness of medications	195	141 (72.3)	42 (21.5)	7 (3.6)	1 (0.5)	4 (2.1)	26.2	10
Setting up meals	196	96 (49.0)	26 (13.3)	5 (2.5)	2 (1.0)	67 (34.2)	25.6	11
Hand washing	195	144 (73.8)	37 (19.0)	8 (4.1)	1 (0.5)	5 (2.6)	24.2	12
Patient teaching	196	148 (75.5)	39 (19.9)	4 (2.0)	1 (0.5)	4 (2.1)	22.9	13
Focused reassessments	194	147 (75.8)	38 (19.6)	2 (1.0)	1 (0.5)	6 (3.1)	21.8	14
Skin/wound care	195	152 (78.0)	29 (14.9)	3 (1.5)	0 (0.0)	11 (5.6)	17.4	15
Medications administered within 30 min before or after scheduled time	196	155 (79.0)	25 (12.8)	6 (3.1)	0 (0.0)	10 (5.1)	16.7	16
IV/central line site care and assessments	196	161 (82.1)	26 (13.3)	5 (2.6)	0 (0.0)	4 (2.0)	16.2	17
Response to call light within 5 min	195	157 (80.5)	22 (11.3)	5 (2.6)	2 (1.0)	9 (4.6)	15.6	18
PRN medication within 15 min	195	163 (83.6)	26 (13.3)	2 (1.0)	0 (0.0)	4 (2.1)	14.7	19
Patient assessments each shift	195	163 (83.6)	16 (8.2)	10 (5.1)	0 (0.0)	6 (3.1)	13.8	20
Discharge planning and teaching	195	168 (86.2)	18 (9.2)	3 (1.5)	1 (0.5)	5 (2.6)	11.6	21
Vital signs assessed as ordered	196	170 (86.7)	17 (8.7)	2 (1.0)	1 (0.5)	6 (3.1)	10.5	22
Bedside glucose monitoring as ordered	195	173 (88.7)	11 (5.6)	4 (2.1)	1 (0.5)	6 (3.1)	8.5	23
Monitoring intake/output	196	176 (89.8)	8 (4.1)	3 (1.5)	1 (0.5)	8 (4.1)	6.4	24

a Proportion of “always,” “frequently,” or “occasionally” out of the total responses, excluding “not applicable” from the denominator.

### Missed nursing care by participant characteristics

The mean differences in missed nursing care according to participants’ characteristics are presented in Table 3. Missed nursing care among nurses differed according to their age, work type, and hospital size. Younger nurses reported a higher mean score for missed nursing care than did older nurses. Nurses working in three-shift rotations perceived more missed nursing care than those who did not. Nurses working at larger hospitals had higher mean missed care scores than those at smaller hospitals.

**Table 3:** Missed nursing care by characteristics of participants.

Variables	Mean (SD)	*t*/*F*	*p*-value
Nurse turnover			
Turnover rate for 6 months		2.75	0.099
0%	1.26 (0.28)		
1–14%	1.33 (0.26)		
15–22%	1.45 (0.36)		
23–50%	1.34 (0.39)		
*N* = 161			
Nurse and work-related characteristics			
Sex		1.38	0.241
Male	1.51 (0.99)		
Female	1.32 (0.30)		
*N* = 192			
Age, years in 2022		4.27	0.040
21–30	1.36 (0.35)		
31–40	1.33 (0.31)		
41–50	1.22 (0.18)		
≥51	1.23 (0.28)		
*N* = 191			
Highest nursing education		1.04	0.356
Associate’s degree	1.34 (0.33)		
Bachelor’s degree	1.34 (0.33)		
Master’s degree or PhD in nursing	1.17 (0.15)		
*N* = 188			
Subjective health status			
Very good	1.34 (0.54)		
Good	1.26 (0.23)		
Fair	1.32 (0.28)		
Poor	1.42 (0.36)		
*N* = 189			
Work type		4.14	0.043
3 shifts rotation	1.35 (0.33)		
Other	1.19 (0.24)		
*N* = 195			
Work experience in current hospitals		0.01	0.940
Under 1 year	1.22 (0.23)		
1 year–under 3 years	1.34 (0.38)		
3 years–under 5 years	1.40 (0.39)		
5 years–under 10 years	1.36 (0.30)		
10 years or over	1.28 (0.25)		
*N* = 192			
Perceived staffing adequacy		1.34	0.249
Very insufficient	1.35 (0.32)		
Insufficient	1.34 (0.29)		
Sufficient	1.24 (0.26)		
Very sufficient	1.34 (0.74)		
*N* = 195			
Type of unit		1.86	0.159
Medical	1.27 (0.25)		
Surgical	1.38 (0.36)		
Medical-surgical	1.33 (0.34)		
*N* = 195			

	*r*	*p*-value
Age	−0.139	0.056
*N* = 191		
Work experience in current hospitals	−0.074	0.309
*N* = 192		
Workload	0.003	0.964
*N* = 194		
Hospital size (beds)	0.232	0.001
*N* = 195		

SD, standard deviation; PhD, Doctor of Philosophy.

### Relationships between nurse turnover and missed care

A multivariate regression analysis was conducted to identify the association between nurse turnover and missed nursing care, controlling for nurse and work-related characteristics (Table 4). Nurses working in units with turnover rates between 15 and 22% reported more missed nursing care [*β* = 0.160, standard error (SE) = 0.081] than those working in units with a 0% turnover rate. Among nurse and work-related characteristics, subjective health status was a significant predictor of missed nursing care. Compared to nurses reporting poor health conditions, those reporting good or fair health conditions reported less missed nursing care (*β* = −0.185, SE = 0.082; *β* = −0.142, SE = 0.070, respectively). Hospital size was positively related to missed nursing care (*β* = 0.0026, SE = 0.003). The regression model explained 10.3% (adjusted *R*^2^ = 0.103, *p*-value = 0.015) of missed nursing care.

**Table 4:** Relationships of nurse turnover with missed nursing care.

Variables	Missed nursing care
	*β* (SE)
Intercept	1.705 (0.285)^**^
Nurse turnover	
Turnover rate for 6 months (ref: 0%)	
1–14%	−0.054 (0.090)
15–22%	0.160 (0.081)^*^
23–50%	0.068 (0.082)
Nurse and work-related characteristics	
Sex (ref: female)	
Male	0.190 (0.171)
Age, years in 2022	−0.010 (0.006)
Highest nursing education (ref: associate’s degree)	
Bachelor’s degree	−0.071 (0.073)
Master’s degree or PhD in nursing	−0.068 (0.153)
Subjective health status (ref: poor)	
Very good	−0.045 (0.108)
Good	−0.185 (0.082)^*^
Fair	−0.142 (0.070)^*^
Work type (ref: other)	
3 shift rotation	−0.055 (0.104)
Work experience in current hospitals	0.004 (0.007)
Workload	−0.010 (0.009)
Perceived staffing adequacy (ref: very sufficient or sufficient)	
Very insufficient	0.007 (0.081)
Insufficient	0.062 (0.083)
Type of unit (ref: medical-surgical)	
Medical	−0.056 (0.073)
Surgical	0.038 (0.067)
Hospital size (per 10 beds)	0.006 (0.003)^*^
Adj. *R*^2^	0.103
*F* test	1.98
*p*-value	0.015^*^
*N*	154

SE, standard errors; PhD, Doctor of Philosophy, ^*^p < 0.05 and ^**^p < 0.01.

## Discussion

This study examined the prevalence of missed nursing care and how nurse turnover affects missed nursing care. Concerning missed care, 5 out of 24 nursing activities had a prevalence of more than 50%. According to a previous study (2), several activities (turning patients every 2 h, ambulation, patient bathing/skin care, and emotional support) among those five activities also had a high prevalence. However, the number of activities with a prevalence of 50% or more in that previous study (2) was lower in the current study. Patient ambulation was a frequently missed care item, which is consistent findings from a previous review (3). Clinical care, including vital signs, glucose monitoring, and monitoring intake/output, was rarely missed, with glucose monitoring consistently attended to in both the current and previous studies (20).

Nearly 30% of the nurses reported “not applicable” to patient bathing/skin care, mouth care, feeding patients, or setting up meals. Basic care is often provided by informal caregivers (e.g., family members) in South Korea (29). Thus, a response of “not applicable” for missed care in these areas might reflect the care by informal caregivers, not nurses. Only 4.6% of nurses reported that emotional support was “not applicable,” making it the fifth most frequently missed care. This implies that while nurses perceive emotional support as their responsibility, they cannot always provide sufficient support. These findings highlight nurses’ perceptions of their scope of care, which can be influenced by the healthcare delivery system and care culture (2).

In the multivariate analysis, nurses working in units with a moderate turnover rate (15–22%) reported a higher mean score for missed nursing care than those working in units with a zero turnover rate. High turnover may lead to frequent adjustments and more time spent with new staff (24), increasing workload and reducing staffing levels (25). This can increase missed nursing care, hinder teamwork, and disrupt communication and relationships between staff (30). Teamwork is a predictor of missed nursing care (22, 31). Lower levels of teamwork, deteriorated by frequent turnover, can lead to missed nursing care. Moreover, frequent turnover can cause nurses to spend less time adhering to clinical safety guidelines as they need to adjust new staff, potentially increasing incidents of missed nursing care (21).

Thus, this study demonstrated how nurse turnover can affect missed nursing care, emphasizing the importance of preventing nurse turnover. Turnover entails more than just replacing individual nursing staff; it also has a significant impact on care processes such as missed nursing care. Therefore, developing relevant strategies is crucial to prevent turnover among nurses in acute care hospitals. Practical strategies, such as new graduate transition programs (32) and adequate nurse staffing levels that consider the training and adjustment of new nurses, can facilitate their integration (3).

An unexpected finding was that no statistical difference was observed between the units with a turnover rate of 23–50% and those with zero turnover. Perhaps in nursing units with a high turnover rate (23–50%), a significant portion of nursing care might be delegated to patients’ families or informal caregivers. If this is the case, nurses do not consider these delegated nursing care activities within their scope of practice. Thus, they answered “not applicable” instead of missed care, indicating no significant relationship between the higher turnover rate and missed nursing care. Future studies should examine this in depth.

Notably, nurses who reported good health had relatively lower levels of missed nursing care than those with poor health. In previous studies, the main contributing factors to missed nursing care were related to nurses’ working conditions or patient characteristics, such as workload and staffing levels (4–6) and acuity (5), respectively. Managerial strategies and policies promoting nurses’ health and a health work environment (33) should be implemented to prevent missed nursing care. Institutional and governmental regulations could also be implemented.

Workload and perceived staffing adequacy were used as control variables in the relationship between turnover and missed nursing care. In previous studies, these two variables were significant contributing factors (4–6). However, they were not associated with missed nursing care in the current study. Nurse staffing was measured according to the nurses’ perceptions, not the absolute numerical values. Previous studies also used this measure but found a significant association with missed nursing care (2, 16). A previous review found that 14 out of 18 studies reported a significant relationship between low nurse staffing levels and higher levels of missed care (3). This relationship should be investigated in future research.

A recent review found that nurse turnover negatively affected nurse and patient outcomes, although this relationship was partially supported (34). The underlying mechanisms can be explained by care processes such as missed nursing care. In this study, nurse turnover had an adverse effect on missed nursing care, which may have been detrimental to patient safety and care quality. Unnecessary healthcare expenditures can compromise care quality (e.g., prolonged length of stay). Thus, this study elucidates the underlying mechanism of turnover and outcome relationships. Future studies should aim to validate these findings.

### Limitations

This study has some limitations. The cross-sectional design limits any causal inferences. Although turnover and missed nursing care dare were sequential, inverse relationships must be considered, such as higher levels of missed nursing care leading to turnover. Longitudinal studies are needed to establish causality. Convenience sampling limits generalizability of the findings to other hospitals in South Korea. Data were collected from general hospitals, and the results may differ in other types of hospitals (e.g., tertiary general hospitals). Additionally, data from self-reported surveys are subject to recall bias and socially desirable response errors. Another limitation is the failure to assess confounding variables such as job satisfaction, income, and work–family conflict. Future studies should employ diverse measurement methods with these confounding variables.

## Conclusion

This study investigated the prevalence of missed nursing care and the impact of nurse turnover on missed care. Nurse turnover was associated with missed nursing care, highlighting the adverse effects of nurse turnover on care processes. Nursing shortage is a global concern. Clarifying the adverse effects of nurse turnover could facilitate the development of policies to prevent nurse turnover. Several countries, including South Korea, have developed policies to retain nurses. The current findings inform policy change, further policy development, and strategies to prevent nurse turnover at institutional and national levels.

## Data Availability

The raw data supporting the conclusions of this article will be made available by the authors, without undue reservation.

## References

[1] Kalisch BJ Williams RA. Development and psychometric testing of a tool to measure missed nursing care. J Nurs Adm. (2009) 39:211–9. doi: 10.1097/NNA.0b013e3181a23cf5 19423986

[2] Cho SH Lee JY You SJ Song KJ Hong KJ. Nurse staffing, nurses prioritization, missed care, quality of nursing care, and nurse outcomes. Int J Nurs Pract. (2020) 26:e12803. doi: 10.1111/ijn.12803 31850645

[3] Griffiths P Recio-Saucedo A Dall'Ora C Briggs J Maruotti A Meredith P. The association between nurse staffing and omissions in nursing care: a systematic review. J Adv Nurs. (2018) 74:1474–87. doi: 10.1111/jan.13564, PMID: 29517813 PMC6033178

[4] Lake ET Riman KA Sloane DM. Improved work environments and staffing lead to less missed nursing care: a panel study. J Nurs Manag. (2020) 28:2157–65. doi: 10.1111/jonm.12970 32017302 PMC7590500

[5] Redfern O.C. Griffiths P Maruotti A Recio Saucedo A Smith GB Missed Care Study Group. The association between nurse staffing levels and the timeliness of vital signs monitoring: a retrospective observational study in the UK. BMJ Open. (2019) 9:e032157. doi: 10.1136/bmjopen-2019-032157, PMID: 31562161 PMC6773325

[6] Simonetti M Cerón C Galiano A Lake ET Aiken LH. Hospital work environment, nurse staffing and missed care in Chile: a cross-sectional observational study. J Clin Nurs. (2022) 31:2518–29. doi: 10.1111/jocn.16068 34723415

[7] Bagnasco A Timmins F de Vries JMA Aleo G Zanini M Catania G. Understanding and addressing missed care in clinical placements—implications for nursing students and nurse educators. Nurse Educ Today. (2017) 56:1–5. doi: 10.1016/j.nedt.2017.05.015 28599196

[8] Ball JE Bruyneel L Aiken LH Sermeus W Sloane DM Rafferty AM. Post-operative mortality, missed care and nurse staffing in nine countries: a cross-sectional study. Int J Nurs Stud. (2018) 78:10–5. doi: 10.1016/j.ijnurstu.2017.09.003 28844649 PMC5826775

[9] Min A Yoon YS Hong HC Kim YM. Association between nurses’ breaks, missed nursing care and patient safety in Korean hospitals. J Nurs Manag. (2020) 28:2266–74. doi: 10.1111/jonm.12831 31350775

[10] Aiken LH Sloane DM Ball J Bruyneel L Rafferty AM Griffiths P. Patient satisfaction with hospital care and nurses in England: an observational study. BMJ Open. (2018) 8:e019189. doi: 10.1136/bmjopen-2017-019189 PMC5781188 29326193

[11] Chapman R Rahman A Courtney M Chalmers C. Impact of teamwork on missed care in four Australian hospitals. J Clin Nurs. (2017) 26:170–81. doi: 10.1111/jocn.13433 27322941

[12] Srulovici E Drach-Zahavy A. Nurses’ personal and ward accountability and missed nursing care: a cross-sectional study. Int J Nurs Stud. (2017) 75:163–71. doi: 10.1016/j.ijnurstu.2017.08.003 28829974

[13] Thomas-Hawkins C Flynn L Dillon J. Registered nurse staffing, workload, and nursing care left undone, and their relationships to patient safety in hemodialysis units. Nephrol Nurs J. (2020) 47:133–42. doi: 10.37526/1526-744X.2020.47.2.133 32343087

[14] Gurková E Mikšová Z Šáteková L. Missed nursing care in hospital environments during the COVID-19 pandemic. Int Nurs Rev. (2022) 69:175–84. doi: 10.1111/inr.12710 34433226 PMC8653289

[15] Blackman I Lye CY Darmawan IGN Henderson J Giles T Willis E. Modeling missed care: implications for evidence-based practice. Worldviews Evid-Based Nurs. (2018) 15:178–88. doi: 10.1111/wvn.12285 29569380

[16] Cho SH Mark BA Knafl G Chang HE Yoon HJ. Relationships between nurse staffing and patients’ experiences, and the mediating effects of missed nursing care. J Nurs Scholarsh. (2017) 49:347–55. doi: 10.1111/jnu.12292 28388827

[17] Chaboyer W Harbeck E Lee BO Grealish L. Missed nursing care: an overview of reviews. Kaohsiung J Med Sci. (2021) 37:82–91. doi: 10.1002/kjm2.12308 33022855 PMC11896454

[18] Shin S Park JH Bae SH. Nurse staffing and nurse outcomes: a systematic review and meta-analysis. Nurs Outlook. (2018) 66:273–82. doi: 10.1016/j.outlook.2017.12.002 29685321

[19] Dirgar E Berşe S Şahin A Tosun B Manuel Levya-Moral J. Presenteeism and missed nursing care: a descriptive, correlational and observational study. BMC Nurs. (2024) 23:652. doi: 10.1186/s12912-024-02253-9 39272086 PMC11401345

[20] Çiriş Yildiz C Değirmenci Öz S Yilmaz Kuşakli B Korkmaz I. The relationship between work environment and missed nursing care in nurses: the moderator role of profession self-efficacy. J Patient Saf. (2024) 20:522–7. doi: 10.1097/PTS.0000000000001266 39190334

[21] Labrague LJ Al Sabei S AbuAlRub R Burney I Al Rawajfah O. The role of nurses’ adherence to clinical safety guidelines in linking nurse practice environment to missed nursing care. J Nurs Scholarsh. (2024). doi: 10.1111/jnu.13017 39160684

[22] Kohanová D Solgajová A Cubelo F. The association of teamwork and missed nursing care in acute care setting: a mixed-methods systematic review. J Clin Nurs. (2024) 33:3399–413. doi: 10.1111/jocn.17182 38661121

[23] Hospital Nurses Association. A survey on hospital nursing staffing 2018. Business report for Hospital Nurses Association. Available at: https://khna.or.kr/home/pds/utilities.php, (Accessed June 14, 2024).

[24] Bae SH Mark B Fried B. Impact of nursing unit turnover on patient outcomes in hospitals. J Nurs Scholarsh. (2010) 42:40–9. doi: 10.1111/j.1547-5069.2009.01319.x 20487185

[25] Park SH Boyle DK Bergquist-Beringer S Staggs VS Dunton NE. Concurrent and lagged effects of registered nurse turnover and staffing on unit-acquired pressure ulcers. Health Serv Res. (2014) 49:1205–25. doi: 10.1111/1475-6773.12158 24476194 PMC4239846

[26] Faul F Erdfelder E Lang AG Buchner A. G*Power 3: a flexible statistical power analysis program for the social, behavioral, and biomedical sciences. Behav Res Methods. (2007) 39:175–91. doi: 10.3758/BF03193146 17695343

[27] Brewer CS Kovner CT Greene W Cheng Y. Predictors of RNs’ intent to work and work decisions 1 year later in a U.S. national sample. Int J Nurs Stud. (2009) 46:940–56. doi: 10.1016/j.ijnurstu.2008.02.003 18377910

[28] Shin S Oh SJ Kim J Lee I Bae SH. Impact of nurse staffing on intent to leave, job satisfaction, and occupational injuries in Korean hospitals: a cross-sectional study. Nurs Health Sci. (2020) 22:658–66. doi: 10.1111/nhs.12709 32144854

[29] Cho SH Kim YS Yeon KN You SJ Lee ID. Effects of increasing nurse staffing on missed nursing care. Int Nurs Rev. (2015) 62:267–74. doi: 10.1111/inr.12173 25762430

[30] Price JL. The study of turnover. Ames, IA: Iowa State University Press (1977).

[31] Bragadóttir H Kalisch BJ Tryggvadóttir GB. Correlates and predictors of missed nursing care in hospitals. J Clin Nurs. (2017) 26:1524–34. doi: 10.1111/jocn.13449 27325454

[32] Rush KL Janke R Duchscher JE Phillips R Kaur S. Best practices of formal new graduate transition programs: an integrative review. Int J Nurs Stud. (2019) 94:139–58. doi: 10.1016/j.ijnurstu.2019.02.010 30965203

[33] Min A Min H Hong HC. Work schedule characteristics and fatigue among rotating shift nurses in hospital setting: an integrative review. J Nurs Manag. (2019) 27:884–95. doi: 10.1111/jonm.12756 30737987

[34] Bae SH. Noneconomic and economic impacts of nurse turnover in hospitals: a systematic review. Int Nurs Rev. (2022) 69:392–404. doi: 10.1111/inr.12769 35654041 PMC9545246

